# Standardized ketogenic dietary preparation for metabolic PET imaging in suspected and known cardiac sarcoidosis

**DOI:** 10.1093/ehjimp/qyae037

**Published:** 2024-05-09

**Authors:** Erika Hutt, Ghazaleh Goldar, Wael A Jaber, Paul C Cremer

**Affiliations:** Department of Cardiovascular Medicine, Cleveland Clinic Foundation, 9500 Euclid Ave, Cleveland, OH, 44195, USA; Department of Cardiovascular Disease, University of Iowa Health Care, Iowa City, IA, USA; Department of Cardiovascular Medicine, Cleveland Clinic Foundation, 9500 Euclid Ave, Cleveland, OH, 44195, USA; Department of Cardiology and Radiology, Northwestern Medicine, Chicago, IL, USA

**Keywords:** cardiac sarcoidosis, F18-FDG PET, dietary preparation, myocardial suppression, ketogenic diet

## Abstract

**Aims:**

A major limitation of cardiac positron emission tomography (PET) with F18-fluorodeoxyglucose (F18-FDG) for the evaluation of cardiac sarcoidosis (CS) is associated with physiologic myocardial glucose uptake. The optimal dietary protocol to suppress physiologic myocardial F18-FDG uptake is not well-established. We aimed to evaluate the diagnostic performance of a novel dietary preparation using a ketone-based infant formula.

**Methods and results:**

Between 2018 and 2021, consecutive studies using a ketogenic dietary preparation were identified (*n* = 198). The rate of non-diagnostic studies due to failure to suppress myocardial glucose was 7.1% (*n* = 14) with a similar incidence in diabetics (*n* = 6, 8.1%). Among studies reported to have no inflammation (*n* = 137), 130 studies (66%) had mean myocardial standardized uptake value (SUV) less than or equal to mean blood pool SUV.

**Conclusion:**

Patient preparation with a ketone-based infant formula resulted in low rate of inappropriate myocardial glucose suppression in patients undergoing F18-FDG cardiac PET to evaluate CS.

## Graphical Abstract

**Graphical Abstract:**
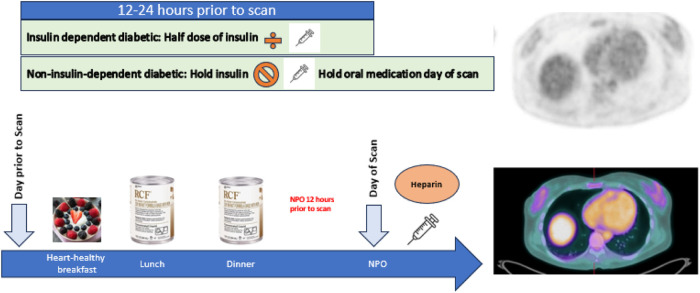
Standardized ketogenic dietary preparation for metabolic cardiac PET imaging. *Dietary protocol description starting the day prior to scan. NPO, nothing by mouth.

## Introduction

Cardiac sarcoidosis (CS) is an infiltrative, inflammatory disease of the heart that can result in ventricular arrhythmias, conduction abnormalities, heart failure, and sudden cardiac death. Diagnosing and evaluating CS is an ongoing challenge due to the heterogenous presentation of disease, low yield of endomyocardial biopsy, and non-specific findings with non-invasive imaging methods. Cardiac positron emission tomography (PET) with F18-fluorodeoxyglucose (F18-FDG) has demonstrated important utility in the diagnosis and monitoring of patients with known or suspected CS and is included in the diagnostic criteria published by the Japanese Circulation Society (JCS) and the Heart Rhythm Society (HRS).^1,2^ The major limitation of this modality is associated with physiologic F18-FDG uptake by normal myocytes.

Under normal circumstances, myocytes utilize a combination of free-fatty acids (FFA) and glucose for energy while inflammatory cells primarily utilize glucose. Thus, to differentiate normal from inflamed myocardium, F18-FDG PET relies on suppression of physiologic F18-FDG (glucose-analogue) uptake.^3^ A variety of dietary preparations have been described to achieve this, all with the objective of inducing ketosis to switch the normal myocardium to utilize FFA. Expert consensus has proposed two possible options for dietary preparation of patients with known or suspected CS prior to F18-FDG PET.^4^ The first includes dietary manipulation with at least two high-fat (>35 g) low-carbohydrate (<3 g) meals the day before the test, followed by 4–12 h fast. For those unable to perform dietary manipulation, 18 h of fasting is recommended as an alternative. While several groups have shown that dietary manipulation is superior to prolonged fasting,^5^ many patients struggle to complete this preparation due to complexity, difficulty with food label understanding, and dietary restrictions, among others.

Since 2018, we developed a novel dietary preparation using a ketone-based infant formula (Ross Carbohydrate Free® by Abbott) in all hospitalized patients undergoing F18-FDG PET for the evaluation or management of suspected or known CS. The aim of this study was to evaluate and report the diagnostic performance of this strategy.

## Methods

This retrospective cohort study included consecutive inpatients between January 2018 and December 2021 who underwent cardiac F18-FDG PET to evaluate CS and received a ketogenic diet preparation with Ross Carbohydrate Free® infant formula. This preparation consisted in the following dietary modification on the day prior to cardiac PET imaging: two meals (lunch and dinner) were replaced by a soy-based infant formula (Ross Carbohydrate Free® infant formula, Abbott), followed by at least 12 h of fasting. In non-insulin-dependent diabetics, instructions were to hold insulin for 12–24 h prior to the test. For insulin-dependent diabetics, instructions were to administer half the dose of insulin for 12–24 h prior to the test. All patients were evaluated for pre-administration of heparin bolus to improve myocardial glucose suppression and all except those with contraindications received heparin. Contraindications to heparin administration included active anticoagulant therapy, recent bleeding event, and heparin allergy. The institutional review board approved this retrospective study and the requirement to obtain informed consent was waived.

### PET acquisition

Cardiac and whole-body PET with computed tomography (CT) were acquired in all studies using resting myocardial perfusion with Rb-82 and metabolic imaging with F18-FDG, on Siemens Biograph mCT PET/CT scanners (Siemens Healthcare, Erlanger, Germany), as previously described.^6,7^ Post-processing and image interpretation were done using 4DM-SPECT software (4DM, INVIA, Medical Imaging Solutions, Ann Arbor, MI) and Syngo Via software (Siemens Healthineers, Erlanger, Germany).

### Data collection

Test results and clinical characteristics were obtained from electronic medical records. The Corridor4DM automatic algorithm^8^ with manual reorientation of F18-FDG PET and Rb-82 PET tomograms was used to retrospectively collect standardized uptake values (SUVs) of the myocardium by two physicians (E.H. and G.G.). Blood pool and liver SUVs were quantified by drawing a region of interest in the left ventricular blood pool and left upper liver segment, respectively. Myocardial SUVs were then utilized to quantify the percentage of FDG uptake and scar (defined as perfusion defect not explained by artefact) in all studies. The percentage of myocardial F18-FDG uptake was quantified using max myocardial SUVs above 1.5 times the blood pool SUV. To evaluate interobserver and intraobserver variability, 10 random studies were remeasured twice by E.H. and G.G. The test results were categorized in three categories: diagnostic with inflammation, diagnostic without inflammation, and non-diagnostic due to inability to suppress physiologic F18-FDG uptake based on clinical reports. A semiquantitative analysis using SUV mean of the myocardium and SUV mean of the blood pool was also developed to classify myocardial F18-FDG uptake. Grade 0 uptake was defined when myocardial SUV mean was lower than blood pool SUV mean, grade 1 uptake when myocardial SUV mean was equal to blood pool SUV mean, and grade 2 uptake when myocardial SUV mean was higher than blood pool SUV mean.

The primary aim was to evaluate the effect of this novel ketogenic diet preparation in F18-FDG PET results, specifically in the rate of non-diagnostic studies. As part of our exploratory analysis, we performed a subgroup analysis among patients with diabetes mellitus. In addition, we sought to determine the rate of non-diagnostic studies in our ambulatory population undergoing standard dietary preparation with high-fat low-carbohydrate dietary modification.

### Statistical analysis

Descriptive statistics for clinical and imaging variables were used. Categorical variables are expressed as absolute number and percentages. Continuous variables are expressed as mean and standard deviation for normal distributions or median and interquartile values for skewed distributions. The Shapiro–Wilk test along with visual plotting using QQ plots was used to assess for sample distribution. Parametric and non-parametric two sample *t*-tests and Pearson χ^2^ tests were used to compare clinical variables and PET results between cohorts. The intraclass correlation coefficient was calculated to assess interobserver and intraobserver variability. R software (version 4.3.1; R Foundation) was used for analyses.

## Results

A total of 1182 F18-FDG PET studies in 846 unique patients were performed at our institution between January 2018 and December 2021 in patients with known or suspected CS. Among these studies, 17% (*n* = 198 among 150 unique patients) were performed on inpatients with a ketogenic diet preparation. All consecutive inpatients were able to tolerate and complete this dietary preparation. *Table 1* shows clinical characteristics and *Table 2* shows imaging characteristics. Patients were middle-aged, and most had a clinical diagnosis of cardiomyopathy. A total of 26 (13%) had known CS, and 172 (87%) had suspected CS. A minority had known diabetes mellitus (*n* = 74). Fasting glucose and fasting time were appropriate as per our local protocol. Important to note that studies reported without inflammation had longer fasting times as compared with those reported as non-diagnostic and with inflammation. A minority of patients were taking prednisone at the time of scan (31%).

**Table 1:** Baseline characteristics of study population stratified by dietary preparation

Variable	Ketogenic diet
	*n* = 198
Age	59 ± 13
Male sex	113 (57%)
White race	136 (69%)
Body mass index (kg/m^2^)	32 ± 8
Hypertension	134 (68%)
Hyperlipidaemia	102 (52%)
Diabetes mellitus	74 (38%)
Atrial fibrillation	92 (47%)
Chronic kidney disease	77 (39%)
Known cardiac sarcoidosis	26 (13%)
Cardiomyopathy	171 (86%)

**Table 2:** PET procedure characteristics and imaging results stratified by dietary preparation

Variable	Ketogenic diet
	*n* = 198
Fasting glucose (mg/dL)	93 (85–111)
Fasting time (hours)	15 (13–18)
Heparin use	103 (63%)
Prednisone use	61 (31%)
LVEF by PET (%)	40 ± 16
EDVI (mL/m^2^)	64 (46–96)
ESVI (mL/m^2^)	39 (23–67)
Max myocardial SUV	3.11 ± 2.42
Mean myocardial SUV	2.02 ± 1.34
Blood pool SUV	1.75 ± 0.59
Liver SUV	0.71 ± 0.96
Liver to SUV myocardial max	0.12 ± 0.28
Blood pool to SUV myocardial max	0.70 ± 0.24
Blood pool to SUV myocardial mean	0.46 ± 0.23
Inflammation (%)	9 ± 23
Scar (%)	11 (15%)
Reported inflammation	48 (26%)
Reported scar	70 (36%)

LVEF, left ventricular ejection fraction; EDVI, end-diastolic volume indexed; ESVI, end-systolic volume indexed; SUV, standardized uptake value.

**Figure 1:**
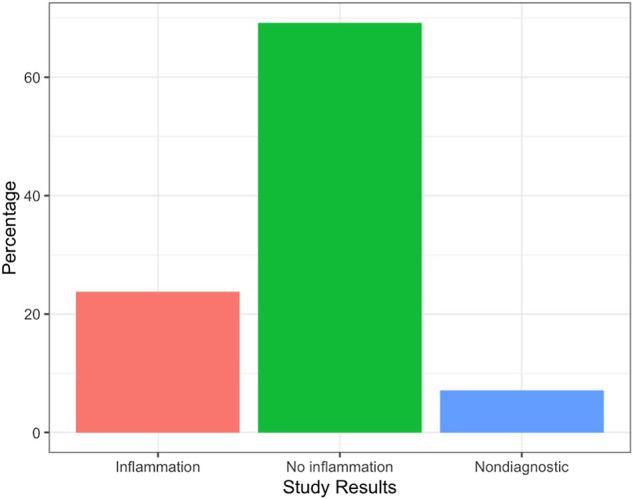
Distribution of F18-FDG PET study results in patients undergoing novel ketogenic preparation.

The reported PET results are shown in *Figure 1*. The overall rate of non-diagnostic PET studies was 7.1% (*n* = 14). The rate of non-diagnostic study results was not significantly higher among diabetics when compared with non-diabetics (8.1% vs. 6.5%, *P* = 0.88).

Regarding the SUV quantification, the overall myocardial F18-FDG uptake quantified using max myocardial SUVs above 1.5 times the blood pool SUV (inflammation percentage) was statistically higher in non-diagnostic studies as expected (*Table 3*). With regard to the semiquantitative classification, a total of 57% (*n* = 113) of studies were classified as grade 0 uptake, 9% (*n* = 17) as grade 1 uptake, and 34% (*n* = 67) as grade 2 uptake. Among studies reported to have no inflammation (*n* = 137), 130 studies (66%) were classified as grade 0 and 1 uptake with mean myocardial SUV less than or equal to mean blood pool SUV.

**Table 3:** Clinical characteristics and F18-FDG PET study findings stratified among non-diagnostic study results

Variable	Non-diagnostic (*n* = 14)	Inflammation (*n* = 47)	No inflammation (*n* = 137)	*P* value
Age	53 ± 14	58 ± 13	60 ± 13	0.13
Male sex	11 (79%)	27 (57%)	11 (79%)	0.23
Body mass index (kg/m^2^)	34 ± 7	30 ± 8	32 ± 8	0.29
Hypertension	11 (79%)	32 (68%)	91 (66%)	0.65
Hyperlipidaemia	6 (43%)	25 (53%)	71 (52%)	0.79
Diabetes mellitus	6 (43%)	20 (43%)	48 (35%)	0.60
Atrial fibrillation	6 (43%)	25 (53%)	61 (45%)	0.57
Chronic kidney disease	2 (14%)	21 (45%)	54 (39%)	0.12
Known cardiac sarcoidosis	1 (7%)	10 (21%)	54 (39%)	0.15
Cardiomyopathy	13 (93%)	45 (96%)	113 (83%)	0.06
Single study	11 (79%)	27 (57%)	112 (82%)	0.01
Fasting glucose (mg/dL)	87 (83–100)	93 (87–105)	93 (85–113)	0.68
Fasting time (hours)	14 (13–20)	14 (13–16)	16 (14–18)	0.01
Heparin use	8 (73%)	27 (66%)	68 (61%)	0.66
Prednisone use	3 (21%)	19 (40%)	39 (28%)	0.23
LVEF by PET (%)	36 ± 16	38 (14%)	41 (17%)	0.42
EDVI (mL/m^2^)	79 (50–116)	74 (57–99)	60 (45–90)	0.25
ESVI (mL/m^2^)	50 (27–77)	45 (29–67)	35 (21–66)	0.29
Max myocardial SUV	5.1 ± 3.3	5.1 ± 3.5	2.2 ± 0.9	<0.001
Mean myocardial SUV	3.5 ± 2.6	2.8 ± 1.7	1.6 ± 0.6	<0.001
Blood pool SUV	1.5 ± 0.4	1.7 ± 0.8	1.8 ± 0.5	0.21
Liver SUV	0.6 ± 0.4	0.9 ± 1.2	0.6 ± 0.8	0.37
Liver to SUV max	0.1 ± 0.2	0.1 ± 0.2	0.1 ± 0.3	0.68
Blood pool to SUV max	0.4 ± 0.2	0.4 ± 0.2	0.8 ± 0.1	<0.001
Blood pool to SUV mean	0.2 ± 0.2	0.2 ± 0.2	0.6 ± 0.2	<0.001
Inflammation (%)	42 ± 44	25 ± 27	0.8 ± 6.1	<0.001
Scar (%)	14 ± 21	14 ± 19	9 ± 12	0.10
Grade				<0.001
0	1 (7%)	5 (11%)	0	
1	1 (7%)	3 (6%)	137 (100%)	
2	12 (86%)	39 (83%)	0	
Reported inflammation	0	47 (100%)	0	<0.001
Reported scar	4 (31%)	23 (49%)	43 (32%)	0.09

LVEF, left ventricular ejection fraction; EDVI, end-diastolic volume indexed; ESVI, end-systolic volume indexed; SUV, standardized uptake value.

Agreement on SUV quantification was good with an intraclass correlation coefficient of 0.939 (95% CI 0.79–0.98) for interobserver agreement and 0.949 (95% CI 0.82–0.99) for intraobserver agreement.

Finally, we found that among ambulatory patients undergoing F18-FDG PET (*n* = 984) for evaluation of CS within the same timeframe of our study population but following the standard of care dietary modification (high-fat low-carbohydrate diet), the rate of non-diagnostic studies was 7.6%.

## Discussion

In this cohort of F18-FDG PET studies in patients with known or suspected CS who underwent ketogenic diet preparation consisting of two-meal substitution with Ross Carbohydrate Free infant formula followed by at least a 12 h fast resulted in an overall rate of non-diagnostic studies of 7%. This finding is important given that implementation of this novel diet is easier as compared with the high-fat low-carbohydrate diet that requires comprehensive caregiver instructions, patient reading and understanding of food labels, and often requires high intake of animal fat that is prohibitive to vegetarians and vegans. This rate was not clinically different than the 7.6% rate of non-diagnostic studies of the ambulatory population at our institution. Moreover, a non-diagnostic rate of 7% is superior to the previously noted acceptable rate of <20%.^3^

Importantly, we found that among diabetics, this dietary modification did not result in a higher rate of non-diagnostic studies. Diabetic patients are known to have impaired myocardial glucose metabolism with use of FFA as the main substrate for energy production that has been reported to result in lower F18-FDG uptake in normal myocardium of patients undergoing viability PET for evaluation of hibernation.^9^ However, after insulin administration myocardial glucose utilization can normalize and switching to FFA utilization can be a challenge. Our dietary preparation along with a reduction or discontinuation of insulin for 12–14 h prior to the test resulted in an acceptable rate of non-diagnostic studies.

Regarding our quantitative assessment, quantification of F18-FDG uptake using SUVs correlated closely with clinical report findings among non-diagnostic studies with higher myocardial SUV max and mean and lower ratios of blood pool SUVs to myocardial SUVs. While visual and qualitative analyses of F18-FDG PET are reflective of current clinical practice, quantitative techniques have the advantage of being more reproducible for assessing the severity (max SUV) and extent (volume of F18-FDG uptake) of inflammation in patients with known or suspected CS.^4^ In addition, the identification of non-diagnostic studies with high quantified ‘volume of inflammation’ in all myocardial territories can be easily evaluated using this quantitative method. Overall, in this study, the reproducibility of SUV analysis is good.

### Limitations

Several limitations of this study should be noted. This is a retrospective study from a single centre without a proper control group for comparison. However, our objective was to evaluate and report the performance and feasibility of this novel ketogenic dietary preparation, which we believe can be widely implemented due to ease of use and patient tolerance. Importantly, only inpatients were included in this feasibility study due to logistical challenges in providing the ketogenic dietary preparation to ambulatory patients. While our findings may not be generalizable to the ambulatory population, we suspect that the inpatient population represents a more challenging cohort to achieve myocardial F18-FDG suppression due to acute illness often accompanied by higher levels of cortisol and glucose; thus, we hypothesize that similar or better results will be obtained in the ambulatory population. Going forward, this important logistical challenge needs to be addressed for generalized use of this ketogenic dietary preparation.

Due to the small number of non-diagnostic studies, multivariable analysis to assess for associations was not possible, although no clinical findings were associated with non-diagnostic studies on univariable analysis. Moreover, while the use of serum beta-hydroxybutyrate (BHB) has been shown to be a highly predictive biomarker for myocardial glucose suppression, routine assessment of serum ketone levels was not performed, and these values may aid in test interpretation.^10–12^ In addition, the lack of an independent and blinded reader may have introduced bias.

Overall, this dietary preparation was straightforward from an ordering provider perspective and while patients did complain of poor taste of the formula, no major adverse reactions or non-compliance were documented by our nursing team. However, patient-reported satisfaction was not collected and would provide invaluable information regarding ease of use.

## Conclusion

In this study, the use of a novel standardized ketogenic formula resulted in a low rate of non-diagnostic studies due to a failure to suppress normal myocardial glucose utilization. Larger studies with a comparable cohort and comparison to other dietary approaches are necessary to determine if this approach should be generalized.

## Consent

Informed consent was waived due to minimal risk in the setting of registry data as per local IRB.

## Data Availability

The data of this manuscript are available on request due to privacy/ethical restrictions.

## Lead author biography

Erika Hutt, MD, is a cardiologist and current cardiovascular imaging fellow at the Cleveland Clinic Foundation. She was born and raised in Costa Rica, where she studied medicine at the Universidad de Costa Rica. Her interest in clinical research led her to pursue medical training in the USA. She completed Internal Medicine residency and Cardiovascular Medicine fellowship at Cleveland Clinic Foundation, as well as a Clinical Research Masters at Case Western Reserve University. Her current area of research interest is in cardiac sarcoidosis and cardiac imaging. During her free time, Erika enjoys cycling and hiking with friends and family.
